# Violet Phosphorene Nanosheets Induced the Death of Ovarian Cancer Cells by Modulating the Vitamin B6 Pathway

**DOI:** 10.3390/molecules30224453

**Published:** 2025-11-19

**Authors:** Xinyi Zhao, Yujing Xu, Zhengyi Liu, Shiling Dai, Miao Qi, Huaiyan Zhang, Jinying Zhang, Dehui Xu

**Affiliations:** 1 State Key Laboratory of Electrical Insulation and Power Equipment, Xi’an Jiaotong University, Xi’an 710049, China; 2 School of Life Science and Technology, Xi’an Jiaotong University, Xi’an 710049, China; 3 Xi’an Cold Plasma Health Technology Co., Ltd., Xi’an 710049, China

**Keywords:** violet phosphorene nanosheets, cancer, metabolic pathway

## Abstract

Cancer remains a significant global public health concern. Numerous challenges still remain in its treatment. Recently, a novel two-dimensional material—violet phosphorene nanosheets (VPNS)—has shown considerable application potential in the biomedical field due to its unique physicochemical properties. The VPNS with a concentration of 41.00 μg/mL has been demonstrated to exhibit significant anti-cancer effects through the induction of apoptosis. The treatment of VPNS was revealed by cell metabolomics analysis to a marked down-regulation of succinate hemialdehyde expression and an up-regulation of pyridoxine levels in cancer cells. These differentially expressed metabolites are closely associated with the vitamin B6 metabolic pathway. In addition, the VPNS has also been demonstrated to exert excellent anti-cancer effects within a living organism by in vivo animal experiments.

## 1. Introduction

Cancer still remains one of the leading causes of mortality worldwide, imposing a significant burden on patients, families, and society at large. However, existing cancer treatment modalities often result in severe adverse effects due to a lack of targeted selectivity, thereby limiting their clinical applicability [1]. Consequently, developing novel therapeutic strategies with high selectivity and precision has become a critical priority in the field of oncology [2,3,4]. In recent years, nanomaterials have demonstrated considerable potential in cancer treatment owing to their unique physicochemical properties, providing innovative solutions to the limitations of conventional therapies and thus attracting widespread attention from the scientific community [5,6,7].

A variety of anti-cancer strategies based on nanomaterials have been developed so far, including cancer imaging, drug delivery systems, and photothermal/photodynamic therapies [8,9,10]. Hollow porous gold nanocages can overcome the limitations of ultrasonic imaging by offering enhanced spatial and temporal resolution in optical imaging [11]. Furthermore, due to their strong near-infrared absorption capacity, these nanocages can release encapsulated doxorubicin (DOX) in a temperature-dependent manner, leading to the death of cancer cells [12,13]. Magnetic iron oxide nanoparticles can also be used to develop innovative approaches for cancer diagnosis and therapy [14]. Additionally, nanomaterials can be conjugated with tumor-targeting ligands such as antibodies, growth factors, and cytokines, enabling targeted drug delivery to cancer cells and minimizing toxicity and adverse effects on healthy tissues [6,15,16,17].

Phosphorus is an essential non-metallic element that is widely present in nature. It exists in various allotropic forms and plays a crucial role in the metabolic processes of lipids, carbohydrates, and proteins within living organisms [18]. Black phosphorus (BP), a layered allotrope of phosphorus, has garnered significant attention in recent years. It possesses a puckered honeycomb structure, with individual atomic layers held together by van der Waals forces [19,20]. BP demonstrated remarkable surface reactivity, making it a promising material for biomedical applications such as cancer therapy [21]. It can directly induce cancer cell death as a nanomaterial with high biological activity [22,23] and promoted in situ apoptosis during photothermal therapy (PTT) [24,25,26]. Moreover, it exhibited excellent drug delivery capabilities, enabling the targeted transport of chemotherapeutic and gene drugs to tumor sites for the treatment of malignant tumors, such as non-small cell lung cancer and prostate cancer [27,28].

Violet phosphorus (VP) is another allotrope of phosphorus. In 2019, Zhang et al. successfully synthesized pure VP crystals, determined its lattice structure to be monoclinic, and confirmed that VP exhibited higher thermal stability compared to BP, making it the most stable phosphorus allotrope currently known [29]. Moreover, violet phosphorene nanosheets (VPNS) obtained through the exfoliation from VP crystals have been proven to have extremely high anti-deformation properties and exhibited excellent mechanical properties, making it one of the two-dimensional nanomaterials with great development potential [30]. Due to the relatively short time since the successful synthesis and reporting of VP, the current application research related to it is still relatively limited when contrasted with other phosphorus allotropes such as BP. Existing studies have shown that VPNS demonstrate potential in the field of cancer treatment through PTT and photodynamic therapy (PDT) [31]. In addition, violet phosphorus quantum dots (VPQD) have been proven to efficiently catalyze the generation of reactive oxygen species (ROS) without external stimulation, exhibiting notable catalytic and therapeutic properties. Nevertheless, it remains uncertain whether VPNS possess inherent biological activity and whether they can effectively suppress cancer cell proliferation through individual treatment—without reliance on external triggers such as light or heat—thus necessitating further investigation. In this study, VPNS of three different sizes were prepared, and their morphology and structure were characterized using scanning electron microscopy (SEM), transmission electron microscopy (TEM), atomic force microscopy (AFM), X-ray diffraction (XRD), and Raman spectroscopy. Subsequently, the biological effects of VPNS were investigated. In vitro cell experiments, including cell viability, proliferation, and Annexin V-FITC-based apoptosis detection, were conducted to determine the inactivation effect of VPNS on A2780 ovarian cancer cells. To elucidate the underlying mechanism, intracellular phosphate concentrations were measured, revealing a significant increase in phosphate levels in cancer cells treated with VPNS. Concurrently, notable alterations in the metabolites of A2780 cells were observed, mainly manifested as the upregulation of the vitamin B6 metabolic pathway. Finally, in vivo experiments have shown that VPNS also exhibited anti-cancer activity within living organisms without inducing significant toxicity or adverse effects.

## 2. Results

**Figure 1:**
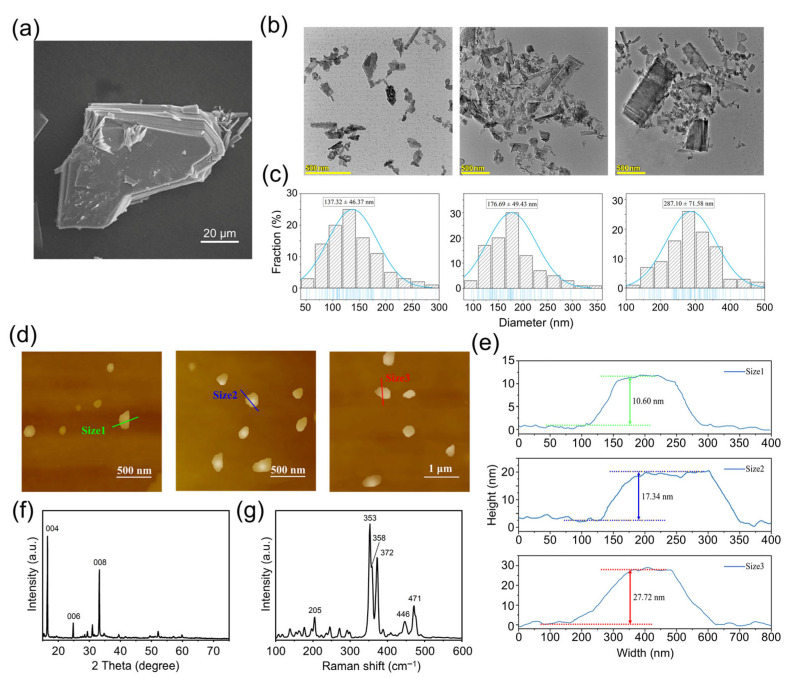
(**a**) SEM image of VP crystals. (**b**) TEM images; (**c**) particle size distribution statistical chart; (**d**) AFM images; (**e**) height statistical chart of exfoliated VPNS; (**f**) XRD pattern; and (**g**) Raman spectroscopy analysis of size 1 (obtained by centrifugation at 8000 rpm), size 2 (5000 rpm), and size 3 (2000 rpm).

The SEM image of the bulk VP crystals is shown in Figure 1a, from which the layer structure can be clearly observed. The observed crystal is sized between 60 μm and 80 μm. Irregular small VP fragments can be observed scattering on the surface of the crystals, which resulted from the fracture of the lamellar structure during the sample preparation process.

The TEM images of VPNS were shown in Figure 1b, and the particle size distribution statistics of three corresponding-sized VPNS were shown in Figure 1c. It can be seen that VP was successfully exfoliated into nanoparticles of three different sizes. The Size 1 VPNS had an average lateral size of 137.32 ± 46.37 nm, Size 2 VPNS were 176.69 ± 49.43 nm in size, and Size 3 VPNS were 287.10 ± 71.58 nm in size. The results indicated that, for the same centrifugation time, the average particle size of the violet phosphorus nanosheets decreased with increasing centrifugation speed. Additionally, higher centrifugation speeds led to a more uniform distribution of nanosheet sizes.

The AFM scanning images of three different-sized VPNS were shown in Figure 1d. The height information of three representative nanosheets was presented in Figure 1e. The representative heights of Size 1, Size 2, and Size 3 VPNS were approximately 10.60 nm, 17.34 nm, and 27.72 nm, respectively. The results suggested that with increasing centrifugation speed, the VPNS not only reduce in lateral size but also decrease in thickness.

The XRD pattern of the violet phosphorus crystal powder is shown in Figure 1f. From the figure, strong, sharp peaks at diffraction angles 2θ of 16.4°, 24.6°, and 33.0° can be observed, corresponding to the {004}, {006}, and {008} reflections of VP. The Raman spectrum of the VPNS sample is shown in Figure 1g. As shown in the figure, vibrational signals corresponding to the P–P bond were observed between 100 and 300 cm^−1^. The Raman peak at 353 cm^−1^ (S^2^ [P9]) corresponded to the stretching vibrational mode of the [P9] cages, while the peaks at 358 cm^−1^ (S^1^ [P8]) and 372 cm^−1^ (S^2^ [P8]) were attributed to the stretching modes of [P8] [32]. Additionally, the peak at 471 cm^−1^ (Tg) was associated with the tangential stretching mode of the [P9] cages along the tubular axis. The results presented in Figure S1 demonstrate that VPNS maintained a negative zeta potential, confirming that the charge carried by the VPNS was negative. The absolute value of zeta potential exhibited a concentration-dependent increase, whereby higher VPNS concentrations corresponded to more negative zeta potential values. Here, a larger absolute value indicated a stronger stability of the VPNS system.

**Figure 2:**
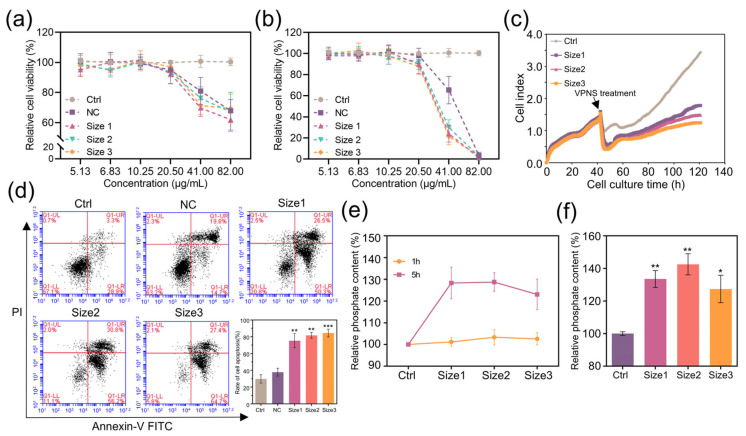
Cell viability after VPNS treatment for (**a**) 3 h and (**b**) 24 h. (**c**) RTCA proliferation curve. (**d**) cell apoptosis detection. The relative phosphate content in the cell (**e**) supernatant and (**f**) intracellular after VPNS treatment. * *p* < 0.05, ** *p* < 0.01, and *** *p* < 0.001.

To explore the anti-cancer effects of VPNS, the VPNS solution was mixed with RPMI-1640 medium at different proportions to generate a series of mixed solutions. The resulting VPNS concentrations were 82.00 μg/mL (20% VPNS), 41.00 μg/mL (10%), 20.50 μg/mL (5%), 10.25 μg/mL (2.5%), 6.83 μg/mL (1.66%), and 5.13 μg/mL (1.25%), with percentages indicating the VPNS proportion in the mixture. A2780 cells were cultured in RPMI-1640 medium containing diverse concentrations of VPNS for 3 h and 24 h. Meanwhile, a negative control (NC) group treated with the same dose of anhydrous ethanol was included to rule out the potential cytotoxic effects of anhydrous ethanol and to accurately assess cell viability. As depicted in Figure 2a,b, VPNS and solvents have little effect on the activity of A2780 cells at 20.50 μg/mL, 10.25 μg/mL, 6.83 μg/mL, and 5.13 μg/mL. At 82.00 μg/mL and 41.00 μg/mL, the activity of cells in the experimental group and the solvent control group declined significantly after 3 h of culture. After 24 h culture, the cell activity in the size 1, size 2, and size 3 groups decreased to 24.2%, 30.0%, and 21.6%, respectively, which was significantly lower than that of the solvent control group. The results demonstrated that VPNS had a killing effect on A2780 cancer cells, and it was time-dependent and dose-dependent. The higher the concentration of VPNS, the longer the treatment time and the stronger its ability to kill cancer cells.

The results of cell activity experiments showed that VPNS and solvent had different cell-killing effects at 41 μg/mL. The micrographs of the cells treated with 41 μg/mL VPNS are shown in Figure S2. To investigate whether VPNS treatment induced cytotoxicity in normal cells, the viability of IOSE80 cells following 24 h exposure to 41 μg/mL VPNS was assessed. As shown in Figure S3, IOSE80 cells treated with three sizes of VPNS showed 70.26%, 67.03%, and 63.05% viability, respectively, which was not significantly different from the NC group. Notably, this stood in sharp contrast to the significant decline in cell viability observed in A2780 cells under identical treatment conditions (41 μg/mL, 24 h). These findings indicated that VPNS selectively targeted ovarian cancer cells at an appropriate concentration, with minimal cytotoxicity to normal cells, thereby demonstrating excellent biosafety.

Subsequently, the effect of varying sizes of 41 μg/mL VPNS on cell proliferation was further examined using a real-time label-free cell analyzer (RTCA). A2780 cells were cultured in E-Plane 96 and subjected to different experimental treatments. Cell culture was then continued, with continuous monitoring of the cell index. The results were presented in Figure 2c. Upon the addition of different sizes of VPNS, the rate of cell proliferation markedly decelerated. After 120 h, the cell index for size 1, size 2, and size 3 groups was 52.35%, 43.52%, and 36.47% of the solvent control group, respectively. These findings reaffirmed that VPNS significantly inhibited the growth of A2780 cells.

To further explore the influence of VPNS on A2780 cells, the apoptosis of A2780 cells treated with 41 μg/mL solvent and various sizes of VPNS was measured via flow cytometry. As depicted in Figure 2d, the apoptosis rate of the VPNS group significantly increased compared with the control group and the solvent group, in which the apoptosis rates of size 1, size 2, and size 3 groups were 75.3%, 81.7% and 84.4%, respectively, indicating that VPNS could mediate the death of A2780 cells by inducing apoptosis. Additionally, the killing effect of VPNS showed a slight enhancement with a larger particle size.

BPNS degrade within cancer cells and result in a rapid escalation of phosphate anion, thereby suppressing the proliferation of cancer cells and inducing apoptosis and autophagy, thereby mediating cell death [21,22]. In order to further explore the mechanism of the anti-cancer effect of VPNS, we measured the change in phosphate content in a 41 μg/mL VPNS solution and A2780 cells after VPNS treatment. After adding solvent and VPNS of different sizes into RPMI-1640 medium, the relative content of phosphate in the solution was detected 1 h and 5 h later, as shown in Figure 2e. It can be perceived that the relative content of phosphate in each group remained relatively invariant after 1 h, and the relative content of phosphate in the VPNS group increased after 5 h. The relative phosphate contents of size 1, size 2, and size 3 groups were 128.3%, 128.8%, and 123.0%, whereas no significant alteration occurred in the solvent control group. Subsequently, solvents and VPNS of different sizes were added to the medium containing the cells, and the relative content of intracellular phosphate was determined 24 h later. The results demonstrated that after 24 h, the intracellular relative phosphate content of the experimental group was conspicuously higher than that of the solvent control group, and the intracellular relative phosphate contents of size 1, size 2, and size 3 groups were 133.5%, 142.5%, and 127.4%, respectively, in Figure 2f. It indicated that VPNS could decompose and produce phosphate ions in culture medium, and concurrently enhance the intracellular phosphate level, exerting a certain influence on the normal growth of cells.

**Figure 3:**
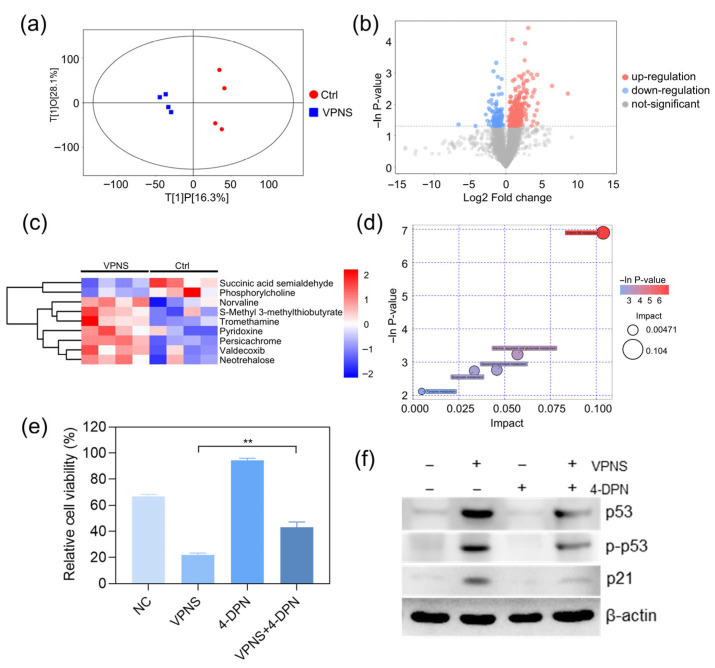
(**a**) Score plot of the OPLS-DA model. (**b**) volcano plot and (**c**) hierarchical clustering heatmap of differentially abundant metabolites. (**d**) metabolic pathway analysis diagram. (**e**) cell viability and (**f**) Western blot of A2780 cells treated with VPNS and 4-DPN. ** *p* < 0.01.

Metabolic reprogramming is a critical hallmark of tumor cells [33,34], and metabolic alterations play a pivotal role in tumor progression. To further elucidate the mechanism underlying the anti-cancer effects of VPNS, we investigated the metabolic differences in A2780 cells between the NC group and the VPNS-treated group using ultra-high-performance liquid chromatography coupled with quadrupole orbitrap mass spectrometry (UHPLC-QE-MS). Figure 3a illustrates the scatter plot of the OPLS-DA model, demonstrating distinct separations between the VPNS-treated group and the NC group. Differential metabolites were visually represented in the form of volcano plots, as shown in Figure 3b. Specifically, the heatmap in Figure 3c reveals that the relative expression levels of succinic semialdehyde and phosphocholine were lower in the VPNS-treated group compared to the NC group, whereas the relative expression levels of Trometamol, pyridoxine, DL-Norvaline, and other substances were higher in the VPNS-treated group than in the NC group. Subsequently, all pathways associated with differential metabolites were identified through KEGG annotation analysis. The five key pathways highest correlated with differential metabolites were further screened, and the results of metabolic pathway analysis were displayed in the form of a bubble chart, as shown in Figure 3d. Notably, the vitamin B6 metabolic pathway exhibited the highest correlation with differential metabolites following VPNS treatment. Additionally, the metabolic pathways of alanine, aspartate, and glutamate in cells were also highly correlated with differential metabolites.

To further elucidate the mechanistic link between the anti-cancer effects of VPNS and the vitamin B6 pathway, we pretreated cells with the vitamin B6 antagonist 4-deoxypyridoxine (4-DPN) prior to VPNS exposure. Subsequently, we assessed changes in cell viability and intracellular protein levels following treatment with 41 μg/mL VPNS. As shown in Figure 3e,f, compared to the direct VPNS treatment group, pretreatment with 4-DPN followed by VPNS significantly rescued cell viability, from 21.97% to 43.07%. Furthermore, we examined the expression of p53 and p21 proteins. Results demonstrated that VPNS treatment markedly increased both the total expression level and phosphorylation status of p53, along with enhancing p21 expression, thereby exerting its anti-cancer effects. However, 4-DPN pretreatment attenuated the VPNS-induced elevation in p53 and p21 expression levels, as well as the phosphorylation activation of p53, suggesting that inhibition of the vitamin B6 signaling pathway compromised the killing effect of VPNS against A2780 cells. Collectively, these findings established that VPNS exerted its anti-cancer effects through activation of the vitamin B6 pathway.

**Figure 4:**
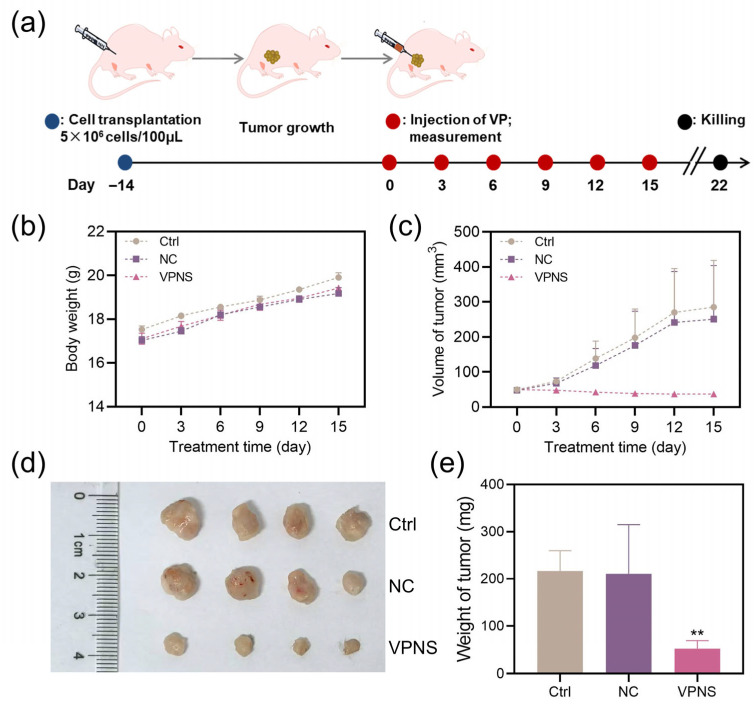
(**a**) Schematic diagram of animal model construction. Changes in (**b**) body weight and (**c**) tumor volume of nude mice after VPNS treatment. (**d**) photos and (**e**) weights of isolated tumors after euthanasia. ** *p* < 0.01.

To further investigate the in vivo anti-tumor efficacy of VPNS, A2780 tumor-bearing nude mice were employed as experimental models, as illustrated in Figure 4a. VPNS were directly injected into the tumor sites of nude mice, and the physiological status and tumor volume were continuously monitored throughout the experimental period. Over the 15-day treatment period, all nude mice exhibited normal dietary, excretion, and activity behaviors, with no mortality or abnormal symptoms observed across any of the groups. The body weight change curve indicated that the average body weight of both the experimental and control groups increased gradually over the 15 days, with comparable growth trends among all groups, as shown in Figure 4b. These findings suggested that VPNS treatment had no adverse effects on the normal physiological conditions of nude mice and did not induce organ toxicity or acute death.

It can be seen from the tumor volume change curve in Figure 4c that the tumor volumes in the control group and the NC group increased significantly. By the 15th day, the average tumor volumes reached 284.82 mm^3^ and 250.84 mm^3^, respectively, indicating that the administration of the solvent alone did not exert any notable tumor-suppressive effect. In contrast, after 15 days of VPNS treatment, a reduction in tumor volume was observed, decreasing from 49.97 mm^3^ to 37.14 mm^3^. This result demonstrated that VPNS exhibit inhibitory effects on tumor growth in vivo, which aligned with the outcomes of the in vitro experiments. Following euthanasia, the tumor tissues were isolated and weighed. As shown in Figure 4d,e, the average tumor weight in the NC group was 210.68 mg, whereas the average tumor weight in the VPNS-treated group was significantly lower at 52.07 mg.

## 3. Discussion

In this study, three VPNS dispersions of varying sizes were prepared via liquid-phase exfoliation. The human ovarian cancer cell line A2780 was selected as the experimental model to investigate the anti-cancer potential of VPNS. Initial cell viability assays confirmed that 0.41 μg/mL VPNS exhibited a significant killing effect on A2780 cells. Although the killing efficiency of VPNS exhibited a marginal increase with increasing VPNS size, the differences among the three sizes were not statistically significant. Furthermore, RTCA cell proliferation detection and Annexin V/PI apoptosis detection verified that VPNS can effectively inhibit cell proliferation and induce apoptosis in ovarian cancer cells. BP was unstable in aqueous media and readily decomposed to form phosphate ions under water-oxygen conditions [35,36]. This degradation process led to a sharp increase in the intracellular concentration of phosphate ions within cancer cells, thereby exerting its anti-cancer activity [22]. So, the intracellular and extracellular phosphate levels following VPNS treatment were also assessed subsequently. Results indicated that VPNS can degrade to release phosphate ions, thereby increasing intracellular phosphate concentrations and disrupting normal cellular physiological functions.

Metabolomic analyses were also performed to compare the VPNS-treated group with the NC group. Through the identification of significantly altered metabolites and key metabolic pathways, the regulatory effects of VPNS on cellular metabolism were elucidated. Vitamin B6 refers to a series of pyridoxoid substances, including pyridoxal, pyridoxol, pyridoxamine, and their phosphorylated derivatives. The phosphorylated forms of vitamin B6 serve as coenzymes for transaminases and decarboxylases, participating in over 140 enzymatic reactions within the body. These reactions are crucial for the metabolism of amino acids, carbohydrates, and fatty acids, as well as for neurotransmitter synthesis [37]. A clinical study on colorectal cancer patients [38] reported that individuals with higher preoperative vitamin B6 levels exhibited improved overall survival rates after diagnosis, suggesting that elevated vitamin B6 status was associated with a reduced risk of colorectal cancer. In addition, several in vitro studies have demonstrated that vitamin B6 can inhibit cell proliferation and enhance the killing effects of chemotherapeutic agents [39]. Research by Zhang et al. [40] revealed that vitamin B6 can activate the p53 signaling pathway in various tumor cell lines, leading to the upregulation of cyclin-dependent kinase inhibitor (p21) expression, which may contribute to the suppression of cell proliferation. Herein, differential metabolite analysis revealed a significantly higher expression level of pyridoxine in the VPNS-treated group compared to the NC group. To clarify the link between VPNS’s anti-cancer effects and vitamin B6 pathway activation, we pretreated cells with 4-DPN (a vitamin B6 antagonist) and measured cell viability and p53/p21 protein levels. The results showed that 4-DPN pretreatment partially reversed VPNS-induced cell viability loss, accompanied by reduced expression and phosphorylation of p53, as well as decreased p21 expression. It suggested that VPNS treatment activated the vitamin B6 metabolic pathway, thereby inhibiting ovarian cancer cell proliferation.

Finally, an A2780 tumor-bearing nude mouse model was established to further evaluate the in vivo anti-cancer efficacy of VPNS. The results showed that VPNS treatment led to a significant reduction in both tumor volume and weight compared to the control and NC groups. Moreover, no abnormal physiological behaviors were observed in the treated mice throughout the experimental period, indicating that VPNS treatment exhibited no significant toxicity or adverse effects.

## 4. Materials and Methods

### 4.1. Preparation of VPNS

The violet phosphorus nanosheets were synthesized by the liquid exfoliation method. In a typical procedure, 100 mg of violet phosphorus crystal powder was dispersed in 200 mL of anhydrous ethanol. The suspension was subjected to ice bath sonication in an ultrasonic cleaner for 48 h and then centrifuged for 10 min to remove the bulk crystals. Violet phosphorus nanosheets of size one, size two, and size three were obtained, respectively, by centrifugation at 8000 rpm, 5000 rpm, and 2000 rpm. The supernatant was then stored in a refrigerator at 4 °C.

### 4.2. Characterization of VPNS

The SEM image was captured using a GeminiSEM 500 field emission scanning electron microscope (Zeiss, Oberkochen, Germany) under an accelerating voltage of 10 kV. TEM and HRTEM images were acquired with a Titan G2 60-300 transmission electron microscope (FEI Company, Hillsboro, OR, USA) with an accelerating voltage of 300 kV. The topology of VPNS was observed by a Bruker Dimension Icon AFM (Bruker, Billerica, MA, USA) under PeakForce Tapping mode. The XRD patterns were obtained using a Bruker D2 PHASER (Bruker, Billerica, MA, USA) using Cu/Kα radiation (λ = 1.5418 Å) at 40 kV and 30 mA. Raman spectroscopy was taken in a backscattering geometry using a single monochromator with a microscope (Reinishaw inVia, Gloucester, UK) equipped with a CCD array detector and an edge filter. The samples were excited by a laser with a wavelength of 633 nm. The zeta potential analyzer (21108415, Malvern Panalytical, Malvern, UK) was used to detect the zeta potential of VPNS.

### 4.3. Cell Culture and Cell Viability Detection

The A2780 ovarian cancer cells used in this study were purchased from the Cell Center of the Chinese Academy of Medical Sciences (Beijing, China). The IOSE80 human normal ovarian surface epithelial cells were purchased from iCell Bioscience Inc. (Shanghai, China). Cells were cultured in RPMI-1640 medium (10-040-CVRC, Corning, Corning, NY, USA) containing 10% Fetal Bovine Serum (FBS) (FBS-E500, NEWZERUM, Christchurch, New Zealand) and 1% Penicillin Streptomycin Solution (HY-K1006, MedChemExpress, Princeton, NJ, USA) at 37 °C in a humidified incubator of 5% CO_2_. To detect the cell viability, A2780 and IOSE80 were seeded in 96-well plates at a density of 8000 cells per well. CellTiter-Lumi™ luminescent cell viability assay kit (C0065M, Beyotime, Shanghai, China) was used to detect the cell activity after experimental treatment. The luminescence value was measured by a microplate reader (Varioskan Flash, Thermo Fisher Scientific, Waltham, MA, USA).

### 4.4. Cell Proliferation Assay

Cell proliferation was measured by the xCELLigence real-time labeled free cell analyzer (xCELLigence RTCA TP, ACEA Biosciences, San Diego, CA, USA). 50 μL medium was added to E-Plane 96 and placed on the RCTA Station for baseline detection to ensure that the Cell Index value of all the holes was below 0.063. Then add 5000 A2780 cells per well and put them into the RCTA Station in the 37 °C incubator for cell culture. The cell index was detected automatically every 15 min. The experimental treatment was carried out 24 h later and then put back into the RCTA Station for culture and continuous cell index monitoring.

### 4.5. Cell Apoptosis Detection

Annexin V-FITC apoptosis detection kit (C1062M, Beyotime) was used to detect apoptosis. Using two fluorescent dyes, Annexin V and PI, the kit uses the Annexin V specificity labeled by green fluorescein FITC to bind to the Phosphatidylserine (PS) that flips outside the cell membrane during apoptosis. PI stains the nucleus to distinguish between normal cells, apoptotic cells, and necrotic cells. Samples were analyzed by a flow cytometer (Accuri C6, BD Biosciences, Franklin Lakes, NJ, USA) within 1 h after staining.

### 4.6. Intracellular Phosphate Detection

Three sizes of VPNS were incubated with both free and cell-containing medium. Add 10 μL Phosphate Sensor (S0192S, Beyotime) of 10 mM and 10 μL sample to each well of the 384-well black plate, mix well at room temperature. Subsequently, use the microplate reader (Varioskan Flash, Thermo Fisher Scientific) for on-board detection. The excitation wavelength is 430 nm, and the emission wavelength is 450 nm.

### 4.7. Cell Metabolomics Analysis

A2780 cells were harvested, and metabolite extraction was subsequently performed. UHPLC-QE-MS analysis was performed using an ultra-high performance liquid chromatograph (Vanquish, Thermo Fisher Scientific) and a combined quadrupole-orbital-trap mass spectrometer (Q Exactive HF-X, Thermo Fisher Scientific). The raw metabolomic data were converted into mzXML format using ProteoWizard software (v3.0). Subsequently, an independently developed R package was used for peak identification, peak extraction, peak alignment, integration, and compound annotation was performed using the self-built secondary mass spectrometry database BiotreeDB (V2.1).

### 4.8. Western Blot Analysis

Cells were lysed in RIPA buffer (containing protease and phosphatase inhibitors) on ice for 30 min, followed by centrifugation at 12,000 rpm for 10 min at 4 °C. The protein concentration was determined using a BCA Protein Assay Kit (DY30205, Deeyee, Shanghai, China). Then separated by SDS–PAGE on 5–12% polyacrylamide gels depending on the molecular weight of the target proteins. Proteins were transferred onto polyvinylidene difluoride (PVDF) membranes (IPVH00010, Millipore, Burlington, MA, USA). After transfer, the membranes were blocked with 5% skimmed milk powder in tris-buffered saline containing 0.1% tween-20 (TBST) at room temperature for 1 h.

The membranes were then incubated with primary antibodies specific to the target proteins at 4 °C overnight. Primary antibodies against p53 (#2524S, 1:1000), phosphorylated-p53 (#9284S, 1:1000), p21 (#2947S, 1:1000) and β-actin (#4970, 1:1000) were purchased from Cell Signaling Technology (Danvers, MA, USA). After washing three times with TBST, the membranes were incubated with horseradish peroxidase (HRP)-conjugated secondary antibodies (1:2000, #7074, Cell Signaling Technology) at room temperature for 1 h. Following additional washes with TBST, protein bands were visualized using an enhanced chemiluminescence (ECL) detection system (DY30208, Deeyee).

### 4.9. Animal Study

In this study, 6-week-old female BALB/c nude mice were utilized as animal models to assess the In Vivo tumor inhibition effect of VPNS. A2780 cell xenografts were established Via subcutaneous injection. Each nude mouse was inoculated with 5 × 10^7^ cells in 100 μL PBS. Two weeks later, the mice were randomly divided into four experimental treatment groups. The control group received no treatment. And the NC group received an injection of 100 μL anhydrous ethanol diluted in PBS, while the experimental group was administered with 100 μL size 1 of VPNS at a concentration of 41 μg/mL. All injections were performed specifically targeting the subcutaneous tumor site. Treatments were administered every three days until day 15. Body weight and tumor volume measurements for each nude mouse were taken prior to each treatment session. On the 22nd day, the nude mice were euthanized, and their tumor tissues were excised and weighed.

### 4.10. Statistical Analysis

Each experiment was repeated three times for reproducibility. Experimental data were presented as mean ± SD. Student’s *t*-test was employed to evaluate the significance between different groups. Differences with * *p* < 0.05, ** *p* < 0.01, and *** *p* < 0.001 were considered statistically significant. All statistical analyses were conducted using GraphPad Prism software (v8).

## 5. Conclusions

We systematically evaluated the anti-cancer effects of three VPNS with different sizes on ovarian cancer cells, both In Vitro and In Vivo. The results demonstrated that at a concentration of 41.00 μg/mL, the VPNS treatment group exhibited a significantly enhanced killing effect against ovarian cancer cells compared to the NC group, with a slight increase in efficacy observed as the size of VPNS increased. Further analysis of cell proliferation and apoptosis revealed that VPNS effectively inhibited cancer cell proliferation and induced apoptosis, thereby contributing to its anti-cancer activity. Additionally, VPNS treatment was found to elevate intracellular phosphate levels. Metabolomic analysis indicated that VPNS induced substantial alterations in the intracellular metabolic profile of ovarian cancer cells. Further metabolic pathway analysis revealed that these differentially expressed metabolites were predominantly associated with the vitamin B6 metabolic pathway, suggesting that VPNS may exert its anti-cancer effects by increasing intracellular phosphate levels and modulating vitamin B6-related metabolic pathways. In Vivo experiments confirmed the anti-cancer potential of VPNS. The VPNS-treated group exhibited a significant reduction in both tumor volume and weight compared to the control group. Importantly, no apparent physiological abnormalities were observed in the VPNS-treated animals, indicating a favorable safety profile at the tested dosage. These findings suggest that VPNS holds promise as a novel therapeutic strategy for cancer treatment.

## Data Availability

Data is contained within the article or Supplementary Material.

## Supplementary Materials

The following supporting information can be downloaded at: https://www.mdpi.com/article/10.3390/molecules30224453/s1. Figure S1. Zeta potential of VPNS dispersed in anhydrous ethanol. Figure S2. Micrographs of A2780 cells treated with 41 μg/mL VPNS. Figure S3. Cell viability of IOSE80 cells treated with 41 μg/mL VPNS.
