# Supplementary material for: Violet Phosphorene Nanosheets Induced the Death of Ovarian Cancer Cells by Modulating the Vitamin B6 Pathway
Source: Molecules. 2025 Nov 19;30(22):4453. doi: 10.3390/molecules30224453 (PMC12655061; doi:10.3390/molecules30224453)
Supplement: Supplementary file 1 [file molecules-30-04453-s001.zip › molecules-3907072-supplementary.pdf]

Violet Phosphorene Nanosheets Induced the Death of Ovarian Cancer Cells by Modulating the Vitamin B6 Pathway

Xinyi Zhao [1,2,†], Yujing Xu [1,2,†], Zhengyi Liu [1,†], Shiling Dai [1], Miao Qi [1,2], Huaiyan Zhang [1], Jinying Zhang [1,*] and Dehui Xu [1,2,3,*]

[1] State Key Laboratory of Electrical Insulation and Power Equipment, Xi'an Jiaotong University, Xi'an 710049, China
[2] School of Life Science and Technology, Xi'an Jiaotong University, Xi'an 710049, China
[3] Xi'an Cold Plasma Health Technology Co., Ltd., Xi'an 710049, China
* Correspondence: jinying.zhang@mail.xjtu.edu.cn (J.Z.); dehuixu@mail.xjtu.edu.cn (D.X.)
† These authors contributed equally to this work.

Supplementary methods of animal study

Nude mice were housed together in individually ventilated cages with four mice per cage. All mice were maintained on a regular diurnal lighting cycle (12:12 light:dark) with ad libitum access to food (XIETONG SHENGWU, Irradiated sterilized propagation feed) and water. Chopped corn cob was used as bedding. Environmental enrichment included nesting material (XIETONG SHENGWU, Nanjing, China), PVC pipe, and shelter (TECNIPLAST, Varese, Italy). Mice were housed in the Medical Animal Center of Xi'an Jiaotong University.

Six-week-old female BALB/c nude mice (16-18g) were randomly divided into three groups (control, NC and VPNS treatment), with four mice per group. Random numbers were generated using the standard = RAND () function in Microsoft Excel.

The study was approved by the laboratory animal care committee of Xi'an Jiaotong University, and was performed according to the committee's guidelines for the use of laboratory animals.

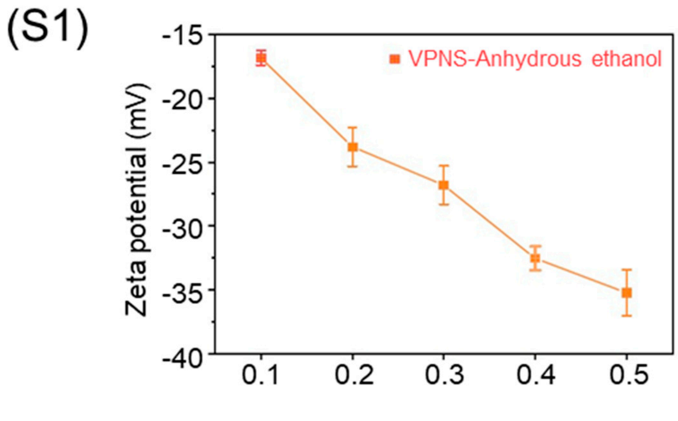

Figure S1. Zeta potential of VPNS dispersed in anhydrous ethanol.

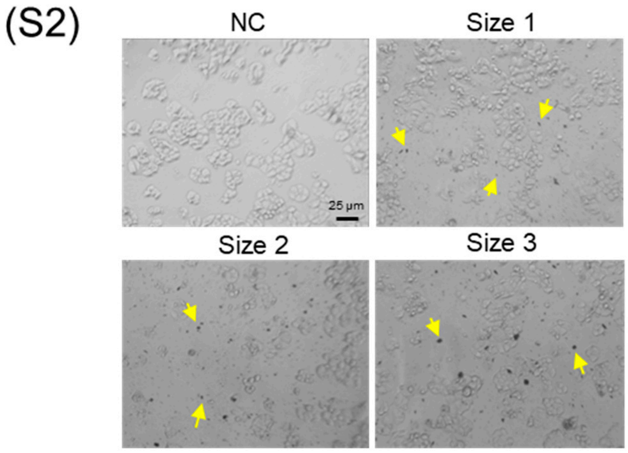

Figure S2. Micrographs of A2780 cells treated with 41μg/mL VPNS.

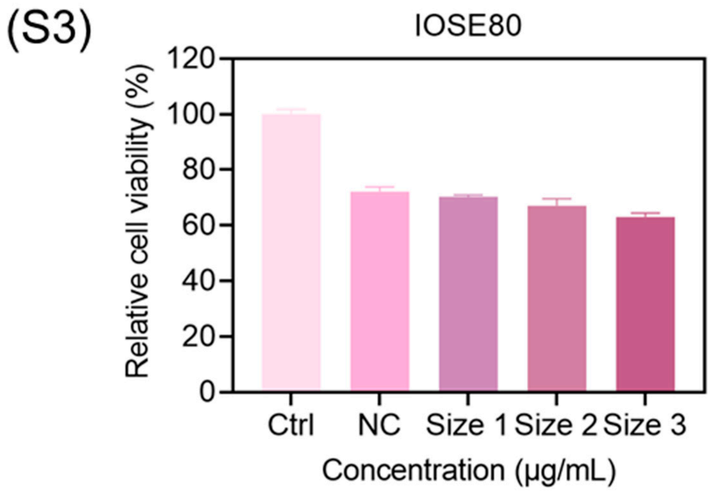

Figure S3. Cell viability of IOSE80 cells treated with 41μg/mL VPNS.
